# Supplementary material for: Single-cell DNA Methylome and 3D Multi-omic Atlas of the Adult Mouse Brain
Source: bioRxiv. 2023 Apr 18:2023.04.16.536509. Preprint. [Version 1] doi: 10.1101/2023.04.16.536509 (PMC10153407; doi:10.1101/2023.04.16.536509)

snm3C-seq FANS gating example

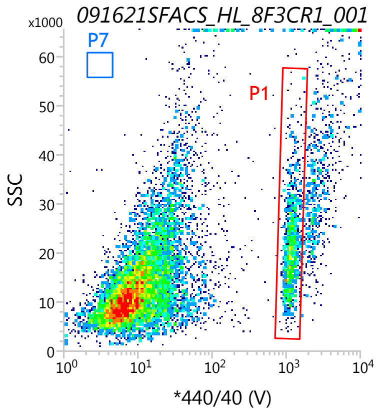

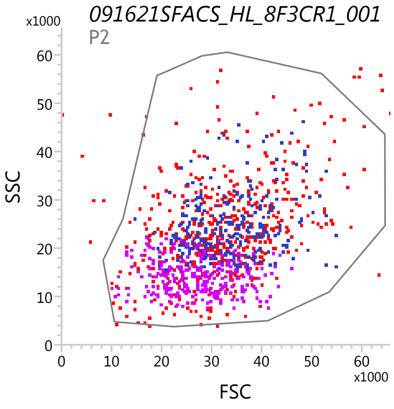

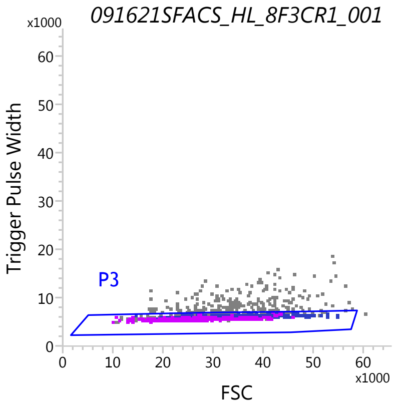

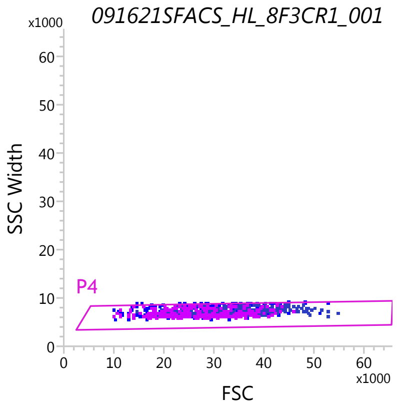

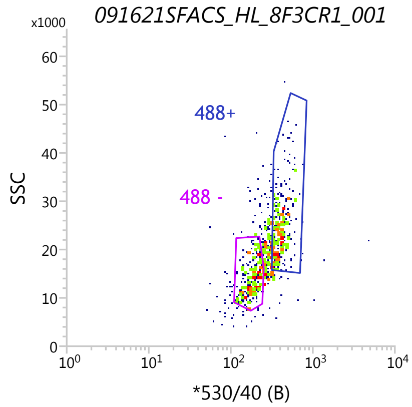

Statistics: *091621SFACS_HL_8F3CR1_001*

Populations	Events	% Total	% Parent
All Events	10,140	100.00%	####
P1	1,049	10.35%	10.35%
P2	1,035	10.21%	98.67%
P3	803	7.92%	77.58%
P4	752	7.42%	93.65%
488+	277	2.73%	36.84%
488 -	267	2.63%	35.51%

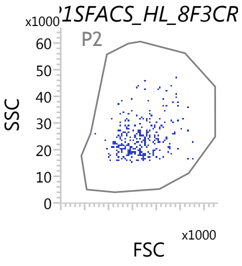

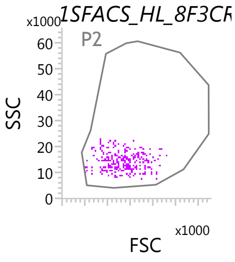

snmC-seq3 FANS gating example

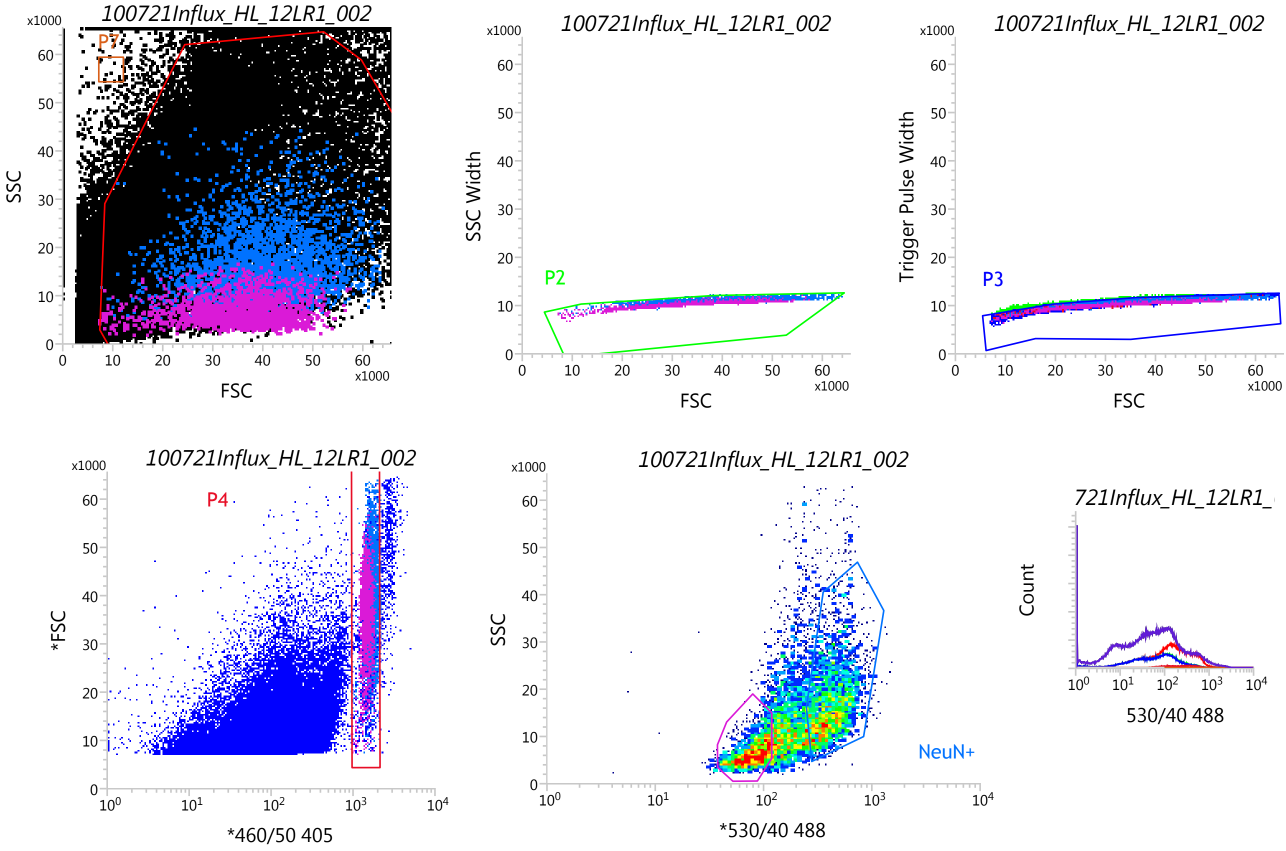

Populations: *100721Influx_HL_12LR1_002*

Populations	Events	% Total	% Parent
All Events	284,075	100.00%	####
P1	116,722	41.09%	41.09%
P2	69,020	24.30%	59.13%
P3	66,582	23.44%	96.47%
P4	7,324	2.58%	11.00%
NeuN+	2,930	1.03%	40.01%
Neg	1,901	0.67%	25.96%
P7	17	0.01%	0.01%
NOT(P7)	284,058	99.99%	99.99%

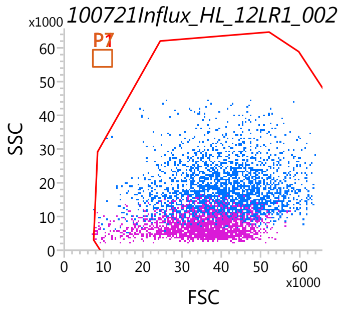

Supplement: Supplement 2 — Supplementary Information 2. FANS gating examples for snmC and snm3C samples. [file media-2.pdf]
